# Cost-Effectiveness of Dronedarone and Amiodarone for the Treatment of Chinese Patients With Atrial Fibrillation

**DOI:** 10.3389/fpubh.2021.726294

**Published:** 2021-08-30

**Authors:** Mengran Zhang, Yu Ren, Luying Wang, Jianhao Jia, Lei Tian

**Affiliations:** ^1^School of International Pharmaceutical Business, China Pharmaceutical University, Nanjing, China; ^2^Center for Pharmacoeconomics and Outcomes Research, China Pharmaceutical University, Nanjing, China; ^3^Pharmaceutical Economics Professional Committee, Hebei Pharmaceutical Association, Hebei, China

**Keywords:** atrial fibrillation, cost-effectiveness analysis, dronedarone, amiodarone, China

## Abstract

**Background:** Atrial fibrillation (AF) is one of the most common arrhythmias in clinical practice, which brings great economic burden to patients. This study evaluated the economics of the new antiarrhythmic drug dronedarone and provides suggestions for allocation of health resources.

**Methods:** Amiodarone was selected as the control group, and the Markov model of AF was established using nine states. The total cost and quality-adjusted life year (QALY) of dronedarone and amiodarone groups were calculated and compared. The incremental cost effectiveness ratio (ICER) value was calculated and compared with the willingness to pay (WTP) and the sensitivity analyses was conducted.

**Results:** For China's healthcare system, the ICER of the dronedarone group compared with the amiodarone group was RMB 81,741 Yuan/QALY, which is lower than the current recommended WTP (3 times GDP per capita). Sensitivity analyses showed that the model was robust, and the drug price of dronedarone significantly impacted the results.

**Conclusions:** Compared with amiodarone, dronedarone is more economical in the Chinese healthcare system. However, due to the lack of data on the Chinese population for some parameters, the model needs further improvement and discussion. Real-world studies on the effects of dronedarone on Chinese patients with AF would be beneficial.

## Introduction

Atrial fibrillation (AF) is one of the most common arrhythmias in the clinic. As of 2017, there were an estimated 37.57 million patients with AF worldwide (1). The lifetime risk of AF in people over the age of 14 years is 26% for men and 23% for women (2). According to an epidemiological survey of 726,451 people in 31 provinces and regions in China in 2017, the standardized incidence of AF among people over 40 years old in China was 2.31%, and with increasing age, the prevalence in women (2.72%) was significantly higher than that in men (1.90%) (3).

The disease burden of AF is heavy, as it may lead to complications such as stroke, thromboembolism, heart failure (HF), myocardial infarction (MI), cognitive decline, dementia, and renal impairment, which can seriously affect patients' quality of life (QoL) and increase their financial burden. The utility value of patients with AF (≥35 years old) measured by the European Five Dimension Health Scale (EQ-5D) scale is 0.53, and the total disability-adjusted life year (DALY) lost due to AF is 665,400 DALY (4).

Zhang et al. (5) found that stroke is the primary cause of the direct economic burden of AF in China, and the treatment cost of stroke caused by AF reaches 4.9 billion RMB every year, of which 89% is from patients with AF and stroke over 60 years old. Therefore, the QoL of patients with AF in China is low, and the economic burden is heavy. Current treatment of AF includes stroke prevention, ventricular rate control, rhythm control, and surgical and hybrid surgical treatment (6). Rhythm control is one of the important strategies for the treatment of AF, and the recovery and maintenance of sinus rhythm is also an indispensable part of the treatment. Currently, commonly used drugs for the maintenance of sinus rhythm include amiodarone, dronedarone, propafenone, sotalol, and flecainide. Among them, dronedarone is used for long-term rhythmic control of paroxysmal and persistent AF. Dronedarone is a new antiarrhythmic drug on the market, which went on the market in 2009. Its chemical structure is similar to amiodarone and clinical trials are numerous. ATHENA trial have shown that dronedarone can reduce the rate of first cardiovascular hospitalization and cardiovascular mortality in patients with non-permanent AF (7). The incidence of the main safety endpoint (MSE: the first occurrence of thyroid-, hepatic-, pulmonary-, neurologic-, skin-, eye-, or GI-specific events, or premature study drug discontinuation following an adverse event) was in the dronedarone group compared with amiodarone group (8).

In recent years, several economic evaluations in various countries have compared dronedarone with other antiarrhythmic drugs (9–13), and most of them have shown that dronedarone is more economical (9–12) than similar drugs for maintaining sinus rhythm. However, no studies on the economic evaluation of dronedarone have been based on the Chinese population; thus, it is unknown if the same conclusions can be applied to China. Therefore, the Markov model was used in this study to evaluate the economic performance of dronedarone in China. Amiodarone was selected as the control group based on the advice and guidelines of clinical experts, quality-adjusted life year (QALY) was used as output, and the incremental cost-effectiveness ratio (ICER) was calculated. The results may provide policy makers and health care providers with practical recommendations to help them make decisions and promote the rational and efficient allocation of health resources.

## Methods

### Overview

We evaluated the cost-effectiveness of dronedarone. The Markov model was used in this study, which can simulate disease progression process through the transfer probability. The outputs in the model were QALY and ICER. The model was conducted from the perspective of the health care system in China. Therefore, only direct health care costs were included and expressed as 2020 values. Both costs and outcomes were discounted by 5% per year (14).

The study group was dronedarone, and the control group was amiodarone. The baseline characteristics of patients were determined based on a multicenter cross-sectional epidemiological survey from the Chinese Atrial Fibrillation Registry published by Sun et al. (15). The initial age was set at a mean age of 68.3 years old, and the female proportion was set at 46.9%.

The Markov model was established in Excel (Microsoft, Redmond, WA, USA) to simulate outcomes and costs. Based on the disease diagnosis and treatment guidelines and the opinions of clinical experts, the model was in a lifetime simulation and the cycle period was set at 1 year.

### Model Structure and Assumptions

The model was based on a simulation of 1,000 individuals in the dronedarone and amiodarone groups. According to “The Understanding and Treatment of AF: Current Recommendations” (6), patients with AF could also face some complications such as HF, stroke, and MI. According to the opinions of clinical experts, the previous pharmacoeconomic evaluation model, the severity and frequency of the complications and the availability of data, other complications were not considered. Therefore, the above mentioned three complications were included in the model, and the additional complications were no longer considered. Most patients with paroxysmal or persistent AF are still at high risk of recurrence after restoration of sinus rhythm, so recurrence was included in the model as a separate state, and the risk of a patient's acute episode (recurrence) was considered in each cycle. Moreover, the adverse reactions of patients were taken into account in each cycle.

**Figure 1:**
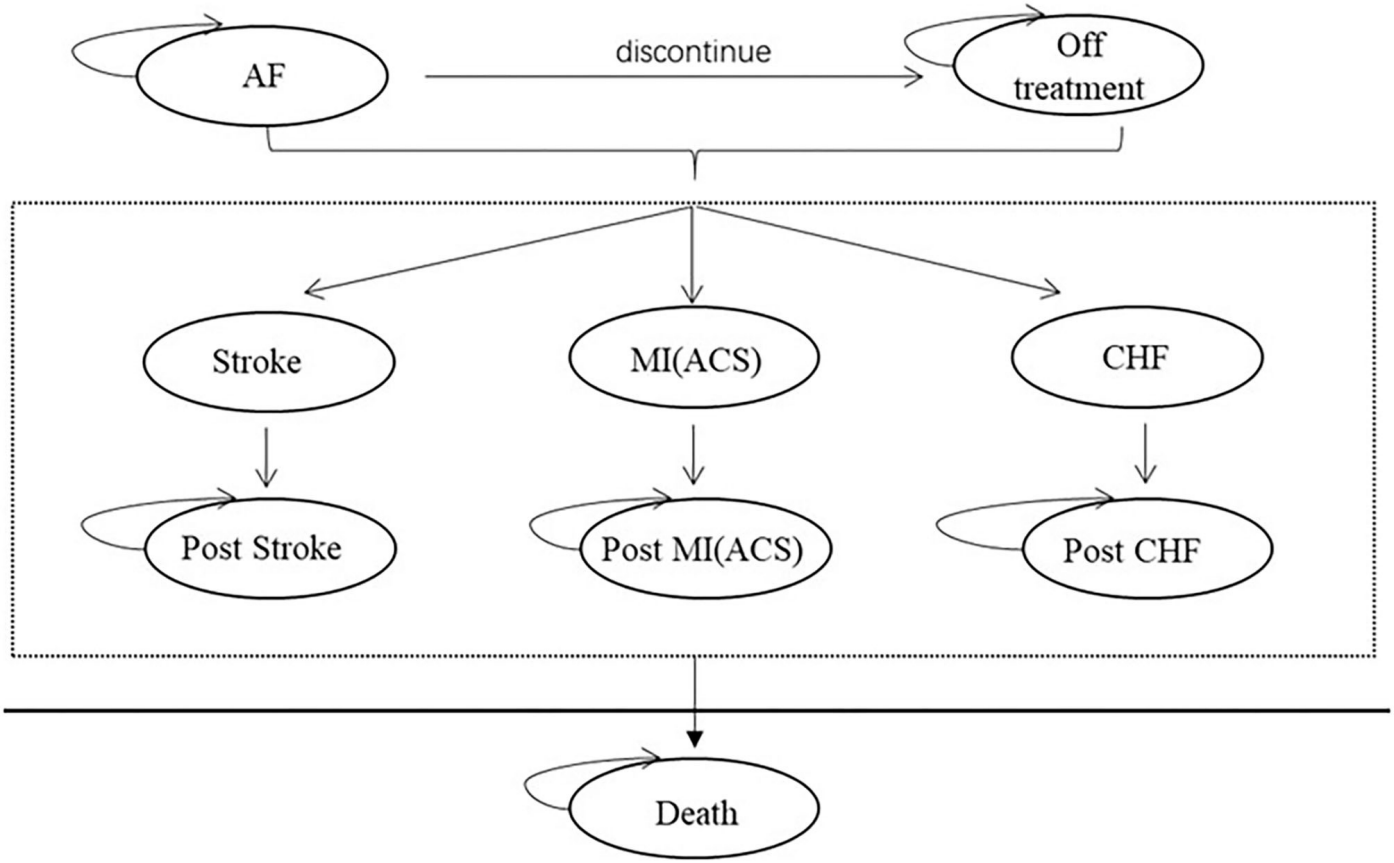
Markov model diagram.

**Figure 2:**
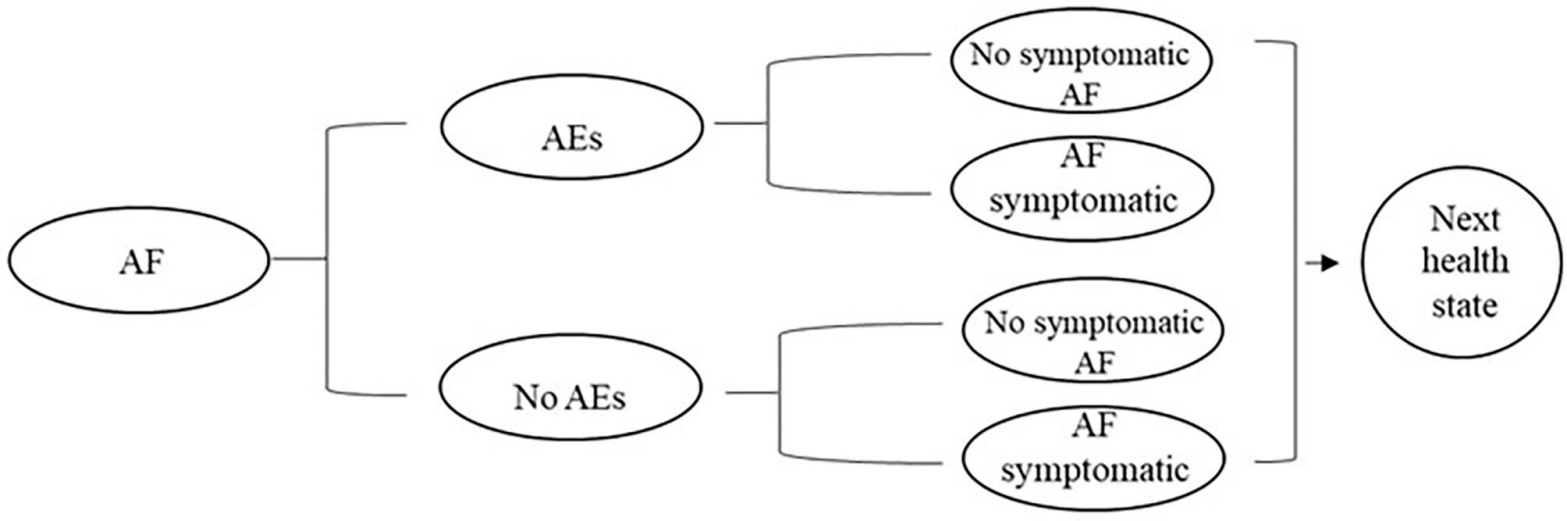
Cycle path within the period.

Based on the existing economic evaluation of AF, the 9-state Markov model was constructed: AF, off treatment, stroke, post-stroke, MI, post-MI, HF, post-HF, and death. The patient enters the model circulation from the state of “AF,” In each cycle, patients with AF are likely to have adverse reactions related to antiarrhythmic drugs. Acute episodes of AF may occur during each cycle in patients with AF, patients in off treatment and patients with each complication. We assumed that only one recurrence of atrial fibrillation occurred per patient per cycle. The model structure and cycle events are shown in Figures 1, 2.

In the process of maintaining sinus rhythm, the treatment plan will be adjusted due to adverse reactions, efficacy, and other reasons. However, according to the guidelines, amiodarone is a relatively backline option, so the model assumed that patients would enter the state of off treatment when they had poor efficacy or intolerance. In addition, the model's assumptions are as follows: (1) Patients will remain in the state of stroke, MI, and HF for one cycle, and then enter the state of post-stroke, post-MI, or post-HF. (2) All disutility and increased utility are calculated one time. (3) Since AF generally does not directly cause death, it is assumed that the mortality of atrial fibrillation is the same as the natural mortality. (4) The costs of rate control drugs were included in off treatment and complication status. Patients discontinued the use of the antiarrhythmic drugs in off treatment, then using rate control drugs. The patients were not taking the rate control drugs while taking the rate control drugs.

### Model Inputs

#### Transition Probabilities

The transition probability between disease states in the model includes drug withdrawal rate related to drug treatment order, the incidence of various types of complications, and mortality. The transition probability parameters are shown in Table 1. The discontinuation rate in the study was based on the annual discontinuation rate of patients in the dronedarone group in the ATHENA study (7), and the discontinuation rate of amiodarone was calculated based on the odds ratio (OR) in an indirect comparative study by Freemantle et al. (16) that included dronedarone, amiodarone, and placebo. When the drug is stopped, the patient will go into off treatment status.

**Table 1:** Values and distribution of model parameters.

**Parameter**	**Base analysis value**	**Lower limit**	**Upper limit**	**Distribution**
**Mortality**				
Stroke vs. natural mortality (RR)	7.4000	6.5000	8.5000	Lognormal
Post-stroke vs. natural mortality (RR)	2.3000	2.0000	2.7000	Lognormal
MI-male vs. natural mortality (RR)	6.1700	5.9800	6.3600	Lognormal
MI-female vs. natural mortality (RR)	8.6700	8.4100	8.9400	Lognormal
Post-MI male vs. natural mortality (RR)	1.4700	1.3900	1.5500	Lognormal
Post-MI female vs. natural mortality (RR)	2.0200	1.9100	2.1500	Lognormal
HF 65–74	0.2200	— —	— —	Beta
HF male > 75	0.3500	— —	— —	Beta
HF female > 75	0.3000	— —	— —	Beta
Post-HF vs. natural mortality (RR)	1.7400	1.3900	2.0900	Lognormal
IPD vs. natural mortality (RR)	2.1000	1.6000	2.7000	Lognormal
ALI	0.0028	— —	— —	Beta
**Complications**				
AF to Stroke: dronedarone	0.0395	0.0237	0.0553	Beta
AF to Stroke: amiodarone vs. dronedarone (HR)	2.0000	1.3000	3.2000	Lognormal
AF to MI: dronedarone	0.0320	— —	— —	Beta
AF to MI: amiodarone	0.0420	— —	— —	Beta
AF to HF: dronedarone	0.1209	0.0895	0.1522	Beta
AF to HF: amiodarone vs. dronedarone (HR)	2.7000	2.0000	3.6000	Lognormal
Off treatment to Stroke vs. AF to Stroke (HR)	1.3514	1.0989	1.6667	Lognormal
Off treatment to MI vs. AF to MI (HR)	1.6949	−0.8621	3.3333	Lognormal
Off treatment to HF vs. AF to HF (HR)	1.2658	1.0417	1.5385	Lognormal
**Off treatment**				
Off treatment: dronedarone	0.1860	— —	— —	Beta
Off treatment: amiodarone vs. dronedarone (OR)	1.0000	0.6300	1.5800	Lognormal
**Recurrence**				
Recurrence: dronedarone	0.6345	— —	— —	Beta
Recurrence: amiodarone	0.4196	— —	— —	Lognormal
Off treatment Recurrence vs. dronedarone (OR)	1.6949	1.3158	2.2222	Lognormal
**Incidence of adverse reactions**				
IPD: dronedarone	0.0219	0.0105	0.0334	Beta
IPD: amiodarone vs. dronedarone (HR)	1.0000	0.5000	2.0000	Lognormal
ALI: dronedarone	0.0076	0.0009	0.0142	Beta
ALI: amiodarone vs. dronedarone (HR)	2.2000	0.8000	6.2000	Lognormal
Hyperthyroidism: dronedarone	0.0080	— —	— —	Beta
Hyperthyroidism: amiodarone	0.0588	— —	— —	lognormal
**Cost (Yuan)**				
Dronedarone	13140	10512	15768	Gamma
Amiodarone	1145	916	1134	Gamma
Examination: dronedarone	2992	2394	3590	Gamma
Examination: amiodarone	2300	1840	2760	Gamma
Anticoagulant	1620	1296	1944	Gamma
Rate control drugs	983	786	1179	Gamma
Stroke	25796	20637	30955	Gamma
Post-stroke	8696	6957	10435	Gamma
MI	65736	52589	78883	Gamma
Post-MI	8544	6835	10253	Gamma
HF	9189	7352	11027	Gamma
Post-HF	3038	2431	3646	Gamma
Recurrence (per time)	213	171	256	Gamma
IPD	21380	17104	25656	Gamma
ALI	9735	3011	16459	Gamma
Hyperthyroidism	5837	4670	7005	Gamma
**Utility**				
AF	0.8100	0.6480	0.9720	Beta
Stroke	0.5600	— —	— —	Beta
Post-Stroke	0.7200	— —	— —	Beta
MI	0.6700	— —	— —	Beta
Post-MI	0.7300	— —	— —	Beta
HF	0.6850	— —	— —	Beta
Post-HF	0.741	— —	— —	Beta
Disutility: recurrence	0.0840	0.0672	0.1008	Beta
Disutility: IPD	0.1900	0.1520	0.2280	Beta
Disutility: ALI	0.1000	0.0800	0.1200	Beta
Disutility: Hyperthyroidism	0.1000	0.0800	0.1200	Beta

*— —, Indicates the missing data; IPD, Interstitial pulmonary disease; ALI, Acute liver injury*.

Due to the lack of complication data in the head-to-head clinical studies of dronedarone and amiodarone, the incidence of stroke and HF was derived from a real-world study published by Gao et al. (17) in 2014 based on a United States healthcare database. The study reported the incidence of stroke, HF, and interstitial lung disease among patients taking antiarrhythmic drugs including dronedarone, amiodarone, and propafenone. The incidence of MI was based on real-world data from a Korean pharmacoeconomic evaluation (13). The incidence of the three complications in the discontinuation status was determined by amiodarone complication rate and was adjusted by rate-controlled versus rhythmically controlled hazard ratio (HR) (18) for complications.

The mortality rate of patients with AF is the same as the Chinese population natural mortality rate Stroke and post-stroke mortality rates were adjusted for the relative risk derived from a long-term survival study of stroke patients (19). MI and post-MI mortality were derived from a Danish study. Mortality in patients with new MI was based on mortality within 1 year of MI, and the mortality of post-MI was based on mortality after 1–3 years (20). The mortality rate of patients with HF was derived from a retrospective study of HF patients in Sweden, and the mortality rate of newly emerging patients with HF was based on the 1-year mortality. The mortality rate of post-HF was derived from a retrospective analysis of the SOLVD trial (21). The relative risk of interstitial pulmonary disease mortality was derived from an 11-year national patient-based study (22). The mortality rate of acute liver injury was derived from a retrospective study in China (23).

#### Recurrence

Relapse parameters are summarized in Table 1. The probability of AF recurrence during treatment was derived from the data of the DIONYSOS trial (8) and the probability of recurrence during off treatment was adjusted by OR from an indirect comparative study by Freemantle et al. (16).

#### Adverse Events

Adverse reaction parameters are shown in Table 1. Based on published economic evaluations, efficacy comparisons, and clinical trial data, the high-risk and clinically severe adverse reactions in the use of dronedarone and amiodarone were considered including interstitial pulmonary disease, acute liver injury, and thyroid dysfunction. The incidence of adverse reactions was derived from Hohnloser et al. (7), Le Heuzey et al. (8), and Gao et al. (17).

#### Cost

The cost parameters are shown in Table 1. Since the research perspective is China's healthcare system, the cost considered in the model was direct healthcare. Based on the clinical treatment needs of patients with AF and the opinions of clinical experts, the direct medical cost in this study included drug cost, cost of routine diagnosis and treatment, cost of treatment of complications, cost of adverse reactions, and cost of treatment for recurrent AF (acute AF episode).

The drugs included in the cost were dronedarone, amiodarone, rate-control drugs, and anticoagulants. The drug price is the median bidding price in the bidding database of Minet. It should be noted that since the original drug of dronedarone has not been used clinically in China, we used the price of the generic version of dronedarone (Daxinning, CSPC Ouyi Pharmaceutical Co., Ltd.). Because there are more amiodarone manufacturers, we weighted the cost of amiodarone according to the market share. Patients who stop treatment will use rate-control drugs whose drug cost is included in the total cost. In addition, a certain percentage of patients with AF receive anticoagulant therapy including warfarin, dabigatran, and rivaroxaban. Based on expert advice, we assumed that 60% of patients received anticoagulant therapy and that warfarin, dabigatran, and rivaroxaban were used at 70, 20, and 10% rates.

The relevant medical items and frequency of use of medical items in the model were determined by “Atrial Fibrillation: Current Knowledge and Treatment Recommendations” (6) and expert consultation. The price of related medical services comes from the medical price documents formulated by the health departments of different cities. In the medical price documents, the median price was taken and weighted according to the proportion of medical institutions of different levels.

Among the costs of complications, stroke and post-stroke costs were derived from an economic evaluation by Ming et al. (24). MI and post-MI costs were derived from economic evaluations of interventional treatment and conservative treatment with drugs of acute non-ST-segment elevation MI based on a Markov model (25). The cost of HF was derived from China Health Statistics Yearbook 2020 (26). Post-HF cost was based on a pharmacoeconomic study published by Sun et al. (27).

Among the costs of adverse reactions, the cost of interstitial lung disease was derived from a study on the etiological classification and disease burden of interstitial lung disease in Fujian, China (28). The cost of acute liver injury was derived from a study measuring the cost associated with drug-induced liver injury, in which we used the cost data of patients hospitalized and whose treatment outcome was improved or cured (29). The cost of hyperthyroidism was derived from China Health Statistics Yearbook 2020 (26).

According to the treatment approach of cardioversion for patients with AF recurrence as stipulated in the guidelines (6), the median price from the medical price documents formulated by the health departments of different cities was taken to calculate the recurrent cost including cardioversion cost, amiodarone treatment cost, consultation fee, bed fee, nursing fee, and intravenous injection fee.

#### Utilities

The utility value parameters are shown in Table 1 and were derived from existing health-related outcome studies of AF or other diseases. The utility value of AF status (without obvious symptoms or complications) was derived from a study of health-related QoL (HRQoL) in 743 patients with AF with an average age of 70.2 years in Taiwan, using the EQ-5D (30). The utility value of patients with AF in the model was 0.81 ± 0.25.

The stroke utility value was derived from a prospective multi-center study conducted by Yeoh et al. (31) in Singapore in 2018. In the study, the changes in health utility values at 3 and 12 months after stroke were −0.25 (−0.18, −0.32) and −0.09 (−0.03, −0.15), respectively. Therefore, the model assumes that the utility value will be reduced by 0.25 and maintained for 1 year after the occurrence of stroke, and the utility value will be reduced by 0.09 at 1 year after the occurrence of stroke (post-stroke state).

The value of MI was derived from a longitudinal study by Munyombwe et al. (32) based on data from the EMMACE-3 and EMMACE-4 trials, which measured the value of EQ-5D-3L scale in patients with AF in the United Kingdom at 1, 6, and 12 months of enrolment. The results showed that the mean utility value was 0.62 (standard deviation: 0.28) during hospitalization and 0.78 at 12 months. At the same time, the average utility value of the British population was 0.86. Based on this, it was calculated that the health utility value of the patients was decreased by 0.14 and 0.08, respectively, after MI and at 1 year after the occurrence of MI. Therefore, in the model, the utility value of patients after MI was decreased by 0.14 and maintained for 1 year, and the utility value after 1 year (post-MI) was decreased by 0.08.

The utility value of HF was derived from Jianwei et al. (33) in a study on the disease burden of Chinese patients with HF, in which EQ-5D-5L was used to measure the QoL of patients. The results showed that the average value of utility in patients with HF was 0.725. Since the utility of the normal population was not reported in the study, the QoL of the elderly (people over 60 years) measured by Yu et al. (34) served as the utility value of the healthy population (0.85 ± 0.20) and made a difference in obtaining the disutility value of HF. Therefore, the disutility value for patients with HF used in the study was 0.125. The value of utility after HF was derived from a study that used EQ-5D to measure the change in health utility value of Swedish patients with HF after 1 year, and the results showed that the health utility value of patients with HF after 1 year was increased by 0.06 compared to baseline (35).

The utility value of interstitial lung disease was derived from a study by Szentes et al. (36), and the utility value of patients with interstitial lung disease was reduced by 0.19. The utility value of thyroid dysfunction was from a health outcomes study based on the Korean population (37). Because utility values for acute liver injury have not yet been retrieved, it was assumed that adverse events would reduce them by 0.1 QALY in patients with AF, based on the study by Nilsson et al. (11).

#### Analytical Methods

First, a basic case analysis was performed, and a deterministic model was run. The values of all necessary parameters were input, the total cost and QALY of the dronedarone and amiodarone groups were calculated and compared, and the ICER value was calculated and compared to the willingness to pay (WTP). The model set WTP as 3 times per capita GDP. According to the statistical data released by the National Bureau of Statistics in 2021, 3 times per capita GDP in 2020 is RMB 217,341 Yuan.

Second, sensitivity analyses were carried out, a one-way sensitivity analyses model and probabilistic sensitivity analysis model were run. In one-way sensitivity analyses, the influence of uncertainty on ICER was tested by changing the value of the parameters. The upper limit or lower limit of the parameter was first determined according to the value and standard deviation in the literature. If neither of the two was determined, the upper limit was assumed to increase by 20%, and the lower limit was assumed to decrease by 20%. The range of the parameters for the one-way sensitivity analysis is shown in Table 1. To further verify the robustness of the model 1,000 Monte Carlo simulations were used to conduct probabilistic sensitivity analyses on the cost and utility, and a scatter plot and cost-effectiveness acceptability curve were drawn.

## Results

### Base-Case Analyses

For the Chinese healthcare system, patients in the dronedarone group received an average of 5.41 QALYs per person during the study period, with a total direct medical cost of RMB 81,862 Yuan per person. Patients in the amiodarone group received an average of 5.14 QALYs per person over the study period, with a direct medical cost of RMB 59,492 Yuan per person.

Compared with amiodarone, the incremental utility of the dronedarone group was 0.27 QALY and the incremental cost was RMB 22,370 Yuan; Compared with amiodarone, patients in the dronedarone group received more health outcomes and spent more, with an incremental cost-effectiveness ratio of RMB 81,740 Yuan/QALY, which is lower than the current commonly recommended WTP (3 times per capita GDP: RMB 217,341 Yuan) in China.

### Sensitivity Analyses

#### One-Way Sensitivity Analyses

**Figure 3:**
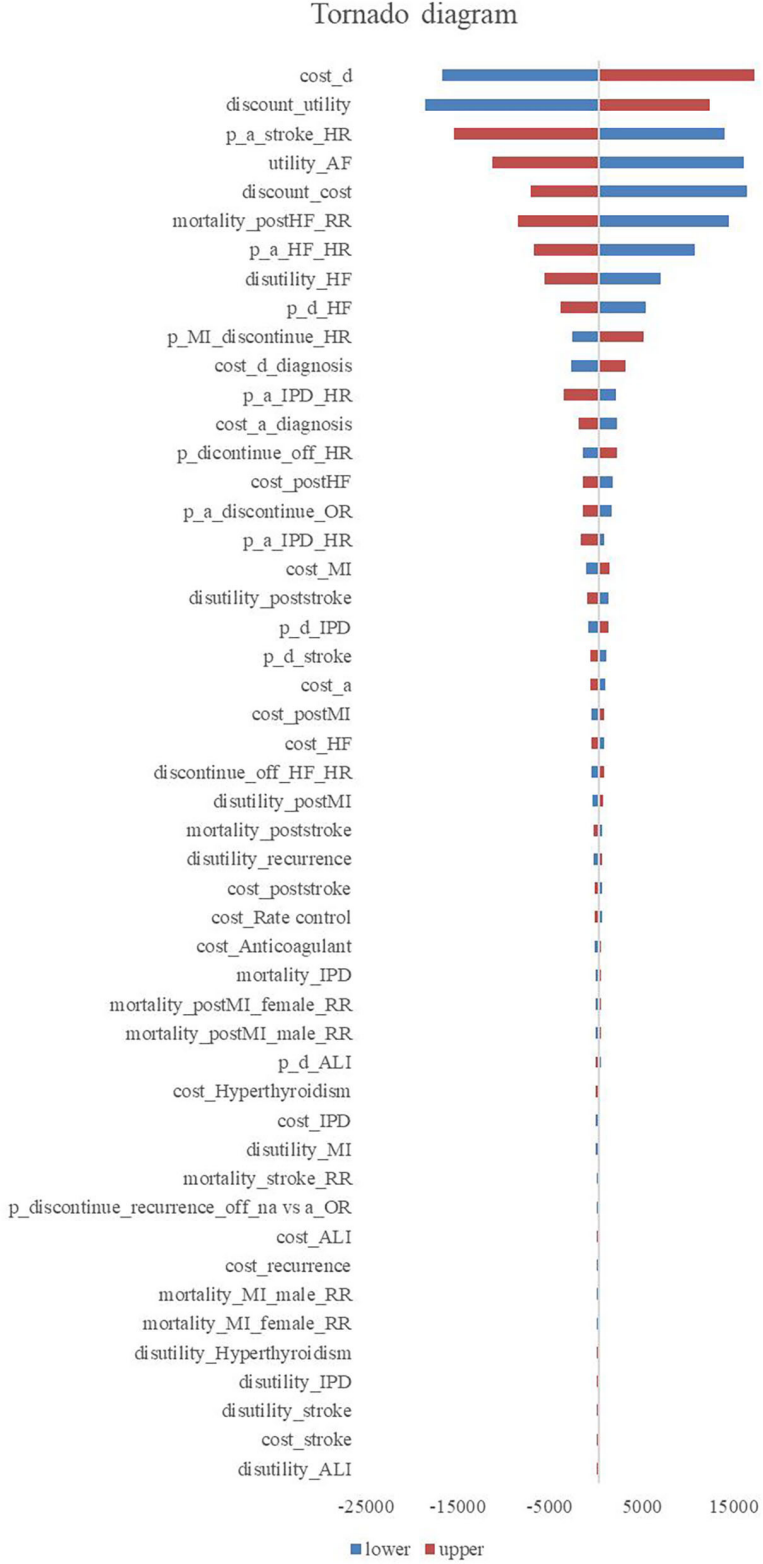
Tornado diagram.

The tornado diagram of one-way sensitivity analysis is shown in Figure 3. According to the results of one-way sensitivity analysis, the drug price of dronedarone, the discount of utility, the HR value of amiodarone vs. dronedarone in patients with stroke, the utility of AF, the discount of cost all greatly influenced the results. The decrease of the drug price of dronedarone and the discount of utility may cause the decrease of ICER. In the other hand, The decrease of the HR value of amiodarone vs. dronedarone in patients with stroke, the utility of AF and the discount of cost may cause the increase of ICER, making dronedarone an uneconomical regimen.

#### Probabilistic Sensitivity Analyses

**Figure 4:**
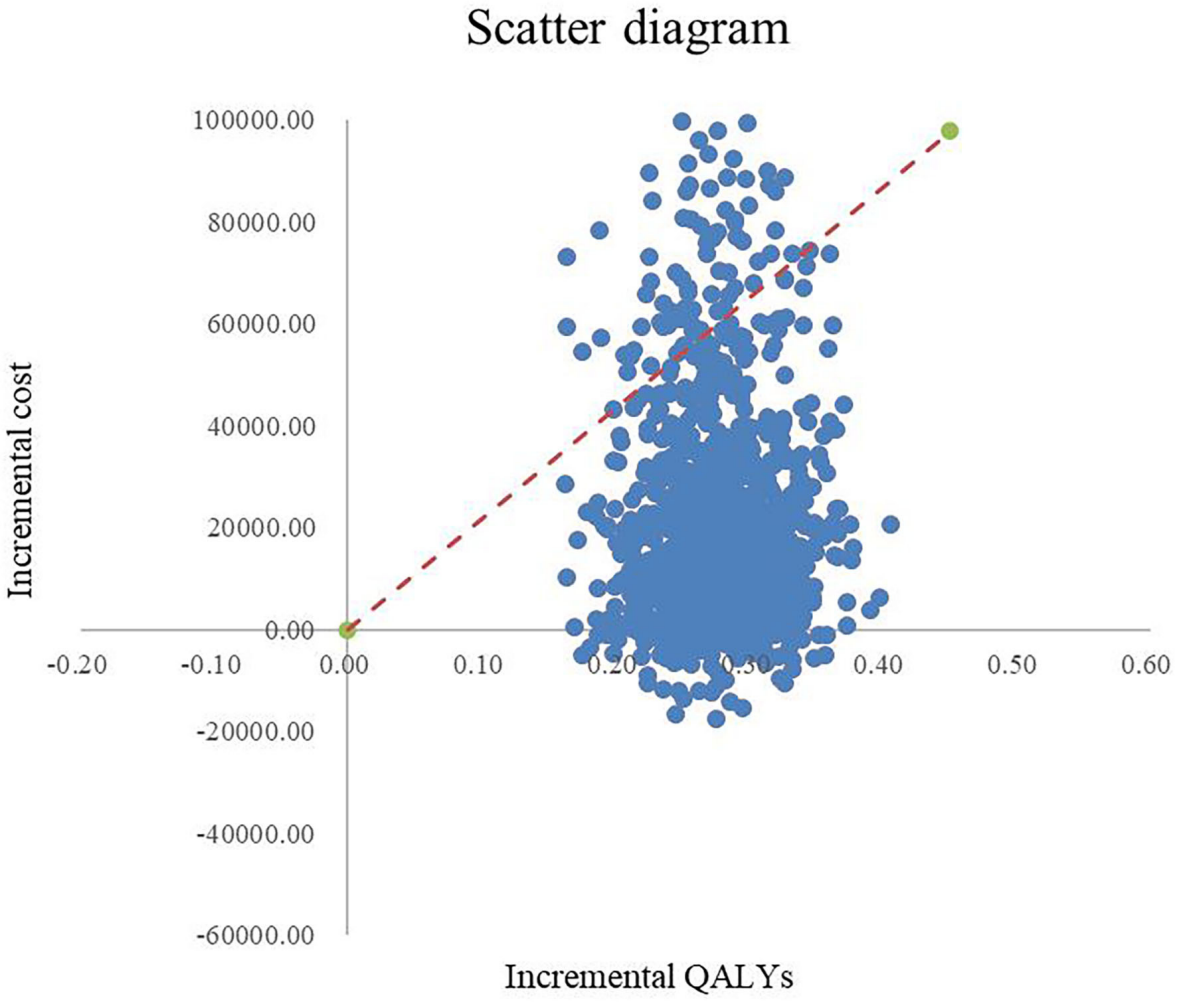
Scatter diagram.

**Figure 5:**
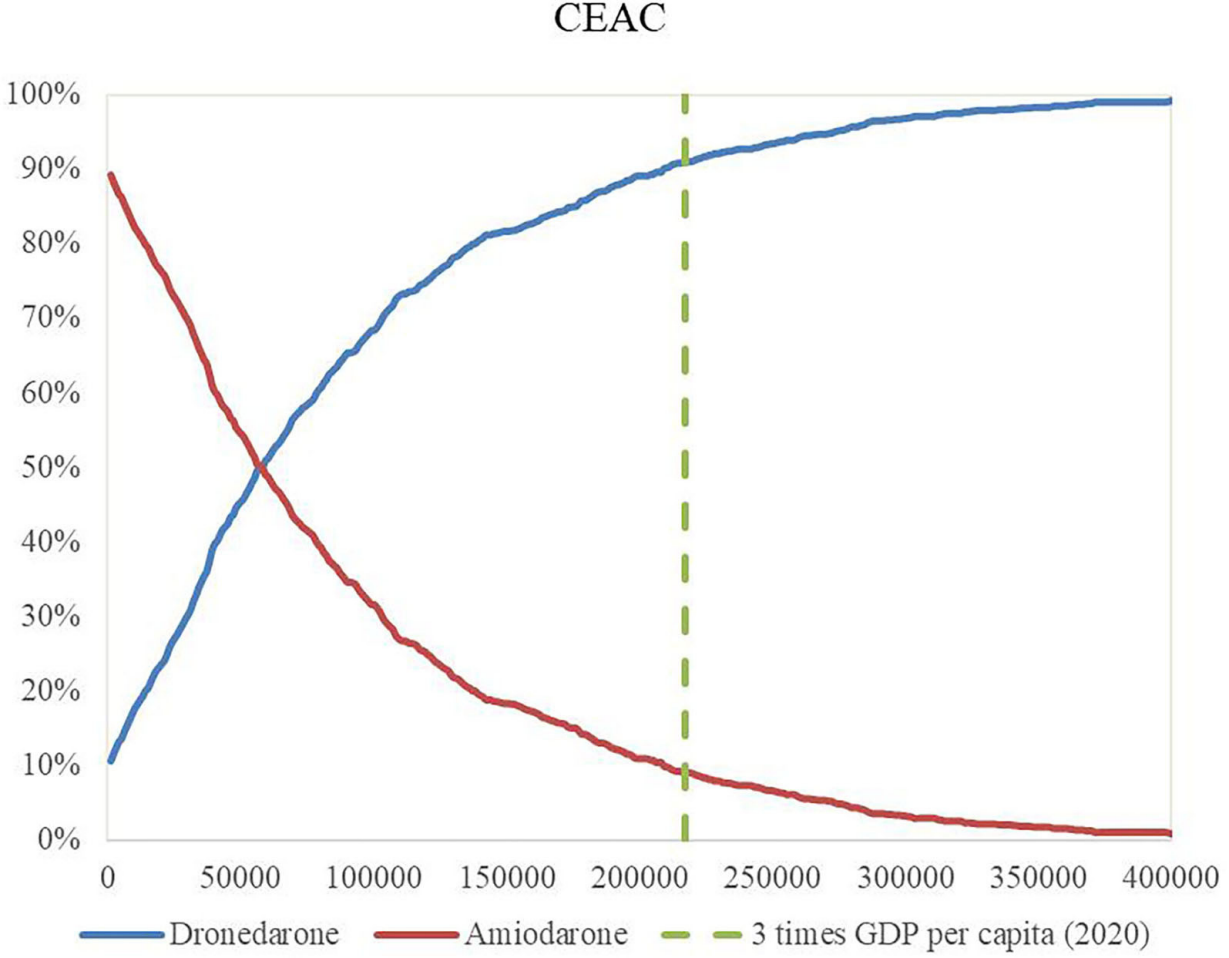
Cost-effectiveness acceptability curve.

Through 1,000 Monte Carlo simulations, we obtained the scatter in Figure 4. Most scatter points are in the first quadrant, which represents dronedarone having higher utility values but also higher costs. The red line in the figure represents the threshold of 3 times GDP per capita, and it can be seen that most of the points were below the threshold, verifying that dronedarone is more economical. The acceptable cost-effectiveness curve is shown in Figure 5, where the horizontal axis represents the range of the WTP threshold. The cost-effectiveness acceptability curve showed that when the WTP was 3 times GDP per capita, the dronedarone plan had an 92% probability of becoming more economical than the amiodarone plan. The stability of the basic analysis results was verified.

## Discussion

This study was the first to evaluate the economics of dronedarone and amiodarone in Chinese patients with AF, which is of significance for the selection of drugs for sinus rhythm maintenance in Chinese patients with AF and provides evidence of resource allocation for government departments. Based on the results of this study, the dronedarone treatment group had higher cost and utility than the amiodarone treatment group, with an incremental cost-effectiveness ratio lower than 3 times GDP per capita currently commonly used in China, and a higher probability of becoming a more economical regimen.

Many studies have been conducted on the economics of dronedarone in various countries using QALY as health outcome. Åkerborg et al. (9) analyzed the economics of adding dronedarone to the standard care of atrial fibrillation from the perspective of health care payers in Canada, Italy, Sweden and Switzerland. The results showed that dronedarone could increase QALYs by 0.10–0.11, and the incremental cost-effectiveness ratio (ICER) of dronedarone per QALY in Canada, Italy, Sweden and Switzerland were 5,828, 5,873, 14,970, and 8,554 euros respectively, which are less than the thresholds of these countries. Uncertainty analysis shows that the use of dronedarone for lifelong treatment and discount rates have a great impact on the research results. Reynolds et al. (12) and Berg et al. (10) conducted the same study from the perspective of health care payers in the United States and Canada, respectively, and obtained similar results. Nilson et al. (11) analyzed the economics of dronedarone and other antiarrhythmic drugs from the perspective of health insurance payers in Canada, Italy, Sweden and Switzerland. The results showed that compared with amiodarone, sotalol and flecainide, dronedarone can increase 0.68–1.90 QALYs, among which, compared with amiodarone, dronedarone can increase 0.86–1.02 QALYs, and ICER value is lower than the threshold of these countries, compared with other antiarrhythmic drugs, dronedarone may be economical. The results of uncertainty analysis showed that RR of mortalities for amiodarone, sotalol, flecainide to dronedarone, discount rates had a greater impact on the research results. Kim et al. (13) analyzed the economics of rhythm control drugs and rate control drugs from the perspective of Korean medical insurance payers, propranolol and pilsicainide were the most economical of rhythm control drugs and rate control drugs, respectively. Uncertainty analysis showed that the basic analysis results were robust. Table 2 showed the detailed information. The basic analysis results of this study showed that, when the simulation time was lifetime, compared with amiodarone, dronedarone produced 0.27 more QALYs, and Similar to the results of other studies in which dronedarone could produce more QALYs. In these countries, changes in cost would make ICER significantly different, and more cost would determine whether dronedarone was relatively economical.

**Table 2:** Information on the economic evaluations of dronedarone.

**Study**	**Country**	**Comparators**	**Perspective**	**Patient population**	**Time horizon**	**Incremental QALYs**	**ICER**	**Major influence factors**
Åkerborg et al. (9)	Canada, Italy, Sweden, Switzerland	Dronedarone + SOC vs. SOC	Health care payer	ATHENA patients	Lifetime	0.10–0.11	€5,828–€14,970	Lifetime therapy with dronedarone, discount rates.
Berg et al. (10)	Canada	Dronedarone +SOC vs. SOC	Health care payer	ATHENA patients	Lifetime	0.13	CAD$7560	Cost of cardiovascular hospitalization.
Nilsson et al. (11)	Canada, Italy, Sweden, Switzerland	Dronedarone vs. amiodarone, flecainide, sotalol	Health care payer	ATHENA patients	Lifetime	0.68–1.9	€2,290–€6,140	RR of mortalities for amiodarone, sotalol, flecainide to dronedarone, discount rates, cost of AF.
Reynolds et al. (12)	US	Dronedarone +SOC vs. SOC	Health care payer	ATHENA patients	Lifetime	0.11	$19,520	Lifetime therapy with dronedarone, no cost associated with AF recurrence on standard of care, discount rates.
Kim et al. (13)	Korea	Rate-control drugs vs. rhythm-control drugs	Health care payer	AF patients who were aged 18 years or older	20 years	2	$1,618	Discount rates, annual drug prices.

*SOC, standard of care*.

From the perspective of clinical efficacy, dronedarone has certain advantages in stroke, HF, myocardial infarction, and other complications as well as thyroid adverse reactions compared with amiodarone, but at the same time, clinical data showed that the RR of dronedarone was higher than that of amiodarone. The RR data were from the DYONISOS trial, but it is important to note that patients in the trial had persistent AF, and according to the applicable scope of the dronedarone, dronedarone was applied to patients with paroxysmal and persistent AF, especially paroxysmal AF. However, due to the uncomprehensive understanding of dronedarone, the trial was not reasonable for the group settings. This may have led to a higher relapse rate among patients in the DYONISOS trial. Due to inclusion of the DYONISOS trial in the meta-analysis of efficacy data and the relatively large sample size, the results of the meta-analysis may be influenced to some extent, which may also lead to a high RR of dronedarone and an underestimation of the health output of dronedarone.

In addition, according to the results of one-way sensitivity analysis, the HR value of stroke incidence in amiodarone patients compared with the dronedarone patients may have a greater influence on the results, which may be related to the higher incidence and mortality of stroke. Moreover, in the basic analysis, the difference of utility value between the two groups was small, making ICER more sensitive to the change of utility value. Similarly, the incidence and mortality of stroke were higher, so the HR of HF incidence in the amiodarone group compared to the dronedarone group may have also influenced the results.

In terms of cost, the annual drug price of dronedarone was much higher than amiodarone. It can also be seen from the results of the basic analysis that the drug cost in the dronedarone group was much higher than that in the amiodarone group, which became the decisive factor of the cost difference. Although the cost in the dronedarone group was reduced in terms of complications and adverse reactions, it was far from enough to offset the difference in drug costs. Based on the current results, a modest reduction in the price of dronedarone would benefit the health benefits of patients with AF and reduce their financial burden.

This study had some limitations. First, due to the lack of complication comparison in the head-to-head clinical studies of dronedarone and amiodarone, the incidence probability of complications included in the model was mainly derived from real-world studies based on the United States population. Therefore, our study had some population heterogeneity issues, which might result in overestimation or underestimation of results. Second, due to the limitation of data and simplifying the model, we assumed that if patients had intolerance or poor curative effect with the current treatment, they would enter a state of drug withdrawal; this setting may have certain differences in clinical practice. However, after two groups of patients are in the same stopped state, the setting for the effect on two groups of patients is the same; thus, when calculating the incremental cost-effectiveness, the effect on the results was limited. At the same time, most of the utility data in the study came from the health output studies of foreign patients, and the situation of QoL in different states in the Chinese population needs to be explored. Finally, the treatment of complications such as stroke, HF, and myocardial infarction in the study was complex, and the cost data were derived from the literature and adjusted for the first year, second year, and beyond. However, this method was not derived from the burden of disease study in Chinese patients and needs to be further optimized in terms of accuracy.

In addition, our study will continue to conduct follow-ups, and the results will be updated according to the follow-up data.

## Conclusions

According to the constructed Markov model, the economic evaluation of Chinese patients with AF receiving dronedarone or amiodarone to maintain sinus rhythm was conducted. The results showed that the incremental cost-effectiveness ratio of dronedarone compared to amiodarone was RMB 81,741 Yuan/QALY, lower than the WTP (3 times per capita GDP) commonly recommended in China at present, which was RMB 217,341 Yuan. The drug price of dronedarone, the HR value of HF in patients with amiodarone compared with dronedarone, and the HR value of stroke in patients with amiodarone compared with dronedarone were significant influencing factors of ICER. When the WTP was three times GDP per capita, the dronedarone plan had an 89% probability of being more economical than the amiodarone.

## Data Availability Statement

The original contributions presented in the study are included in the article/supplementary material, further inquiries can be directed to the corresponding author/s.

## Author Contributions

LT, LW, and JJ designed this study, supervised the data collected, and proposed suggestions for revising the manuscript. Literature analysis were done by MZ, YR, and LW. MZ, YR, and LW built the model using software, collected the data for model, and operated model. MZ drafted the manuscript. All authors critically reviewed the manuscript and approved the final version of the manuscript. All authors agree to be accountable for the content of the work.

## Conflict of Interest

The authors declare that the research was conducted in the absence of any commercial or financial relationships that could be construed as a potential conflict of interest.

## Publisher's Note

All claims expressed in this article are solely those of the authors and do not necessarily represent those of their affiliated organizations, or those of the publisher, the editors and the reviewers. Any product that may be evaluated in this article, or claim that may be made by its manufacturer, is not guaranteed or endorsed by the publisher.
